# Identification of a candidate alternative promoter region of the human Bcl2L11 (Bim) gene

**DOI:** 10.1186/1471-2199-9-56

**Published:** 2008-06-12

**Authors:** Margherita Gaviraghi, Andrea Caricasole, Chiara Costanzo, Daniela Diamanti, Mario Dandrea, Massimo Donadelli, Aldo Scarpa, Marta Palmieri

**Affiliations:** 1 Department of Pathology, Section of Pathological Anatomy, University of Verona, Verona, Italy; 2 Siena Biotech SpA, Siena, Italy; 3 Department of Morphological and Biomedical Sciences, Section of Biological Chemistry, University of Verona, Verona, Italy

## Abstract

**Background:**

Despite the importance of the BCL2L11 (BIM) protein in various apoptotic processes in development and disease, little is known of the promoter structure of the human BCL2L11 locus and of the *cis*-acting elements regulating expression of the human gene.

**Results:**

In the search for novel promoter sequences in the human BCL2L11 locus, we have identified previously unrecognized genomic sequences displaying promoter activity and E2F responsiveness, and driving the expression of BCL2L11 coding transcripts. In man, transcripts originating from this novel putative promoter contribute significantly to total BCL2L11 mRNA expression in testis, heart and liver. In HEK293 cells, this novel candidate promoter originates BCL2L11 transcripts whose expression can be modulated by a known modulator of BCL2L11 expression (Trichostatin A) and by E2F, a characterized transcriptional regulator of BCL2L11 expression.

**Conclusion:**

The identification of a novel putative human BCL2L11 promoter provides new insights into the structure and regulation of the BCL2L11 locus.

## Background

The human BCL2L11 locus, located on chromosome 2q13, encodes a protein of 198 amino acids structurally and functionally related to the BH3-only group of pro-apoptotic BCL2 family members [3]. The gene is frequently mutated in diverse human tumours leading to loss of BCL2L11 activity [14,21]. Expression of BCL2L11 is induced by a diverse range of apoptotic stimuli such as deprivation of growth factors/cytokines, ionizing radiation, and cytotoxic peptides [2,6,14,22]. Although the regulation of BCL2L11 activity is highly complex and is cell context-dependent, studies aimed at identifying the molecular mechanisms underlying BCL2L11 activation during apoptosis have revealed an important role for the transcriptional regulation of BCL2L11 gene expression [1,6,9,12,13,22,23]. A genomic region displaying promoter activity has been characterized for the human BCL2L11 locus [13], while in the rat the existence of three alternative promoter sequences has been postulated, the most upstream of which corresponds to the promoter described for the human BCL2L11 gene [2,3]. Regulation of this conserved BCL2L11 promoter by FOXO and E2F has been described using rat BCL2L11 genomic constructs, thus providing a likely mechanism for the induction of BCL2L11 expression during programmed cell death [2,9], through the involvement of these transcription factors whose activity is induced in apoptotic contexts. Whether regulation by E2F and FOXO factors is conserved in humans remains to be demonstrated; however, such studies are hampered by the relatively poor inter-specific sequence conservation in non-coding sequences and the paucity of information on the structure and regulation of the human BCL2L11 locus. We therefore set out to investigate the existence of as yet uncharacterized human BCL2L11 promoter regions, and to determine the conservation from rodents to man of E2F regulation of BCL2L11 expression.

## Results

### Identification of a novel putative BCL2L11 promoter region and associated BCL2L11 exon

**Figure 1:**
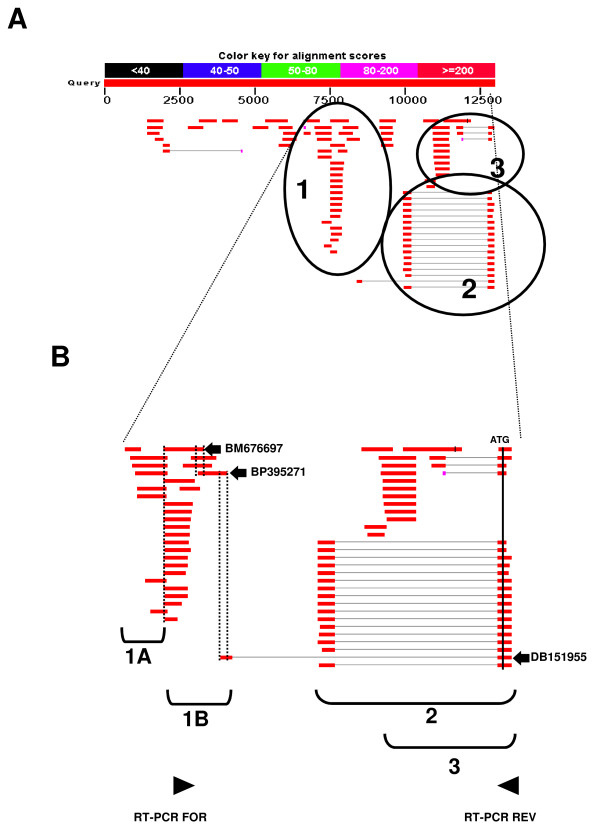
**Bioinformatics analysis recognizes known HsBCL2L11(BIM) promoters and identifies a putative promoter region upstream of these**. **A**. BLAST analysis of 13 kb of genomic sequence upstream of HsBCL2L11 ATG codon (NT_022135.15|Hs2_22291, bases 576452 to 505224) vs Genbank ESTs identified transcribed regions upstream of BCL2L11 locus. Three EST groups can be identified. Of these, groups 2 and 3 correspond to transcripts associated with published BCL2L11 promoters (2: human P2; ref. 13; 3: rat P3, ref. 9). Group 1 is not associated with any published vertebrate BCL2L11 promoter sequences. **B**. Close-up of a ca. 5000 bp region comprising Group 1, 2 and 3 ESTs. The location of the BCL2L11 ATG codon is indicated. One Group 2 EST (DB151955), comprising the first BCL2L11 coding exon, overlaps with Group 1, subgroup B ESTs. Arrowheads indicate the position of RT-PCR primers used to confirm the co-linearity of Group 1, subgroup B ESTs with the first BCL2L11 coding exon.

**Figure 2:**
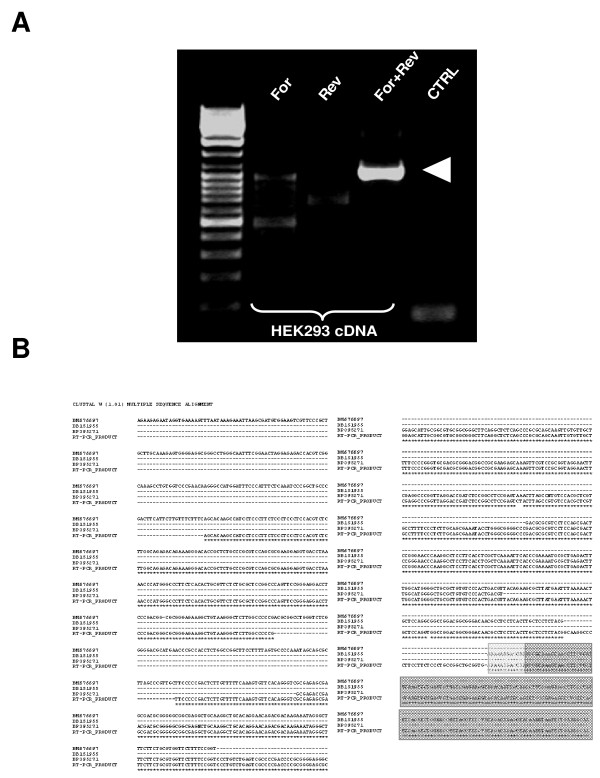
**A transcript originating from the BCL2L11-P1 region in HEK293 cell is a novel BCL2L11 transcript**. **A**. RT-PCR in HEK293 cDNA using a Forward primer anchored within Group 1, subgroup B ESTs and a Reverse primer anchored downstream of the translation initiation codon of HsBCL2L11; a ca. 1000 bp fragment (arrow) is amplified from HEK293 cells. For or Rev: single primer controls; CTRL refers to the no cDNA control. No amplification was observed when RNA was used as template (data not shown). **B**. Sequence of the amplified cDNA fragment and its alignment with ESTs from Group 1, subgroup B (BM676697, BP395271) and from Group 2 (DB151955). The first BCL2L11 coding exon is shaded in grey (BCL2L11 coding sequence in dark grey).

**Figure 3:**
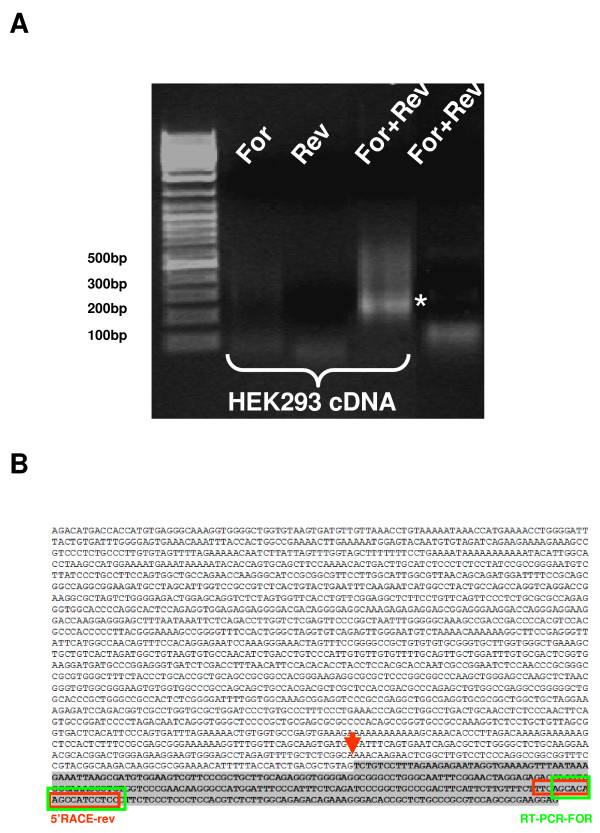
**A novel putative promoter is identified upstream of known BCL2L11 promoters**. **A**. 5'-RACE on transcribed sequences in BCL2L11-P1 to identify transcript initiation sites in HEK293 cells; the major PCR product, indicated by the asterisk, was sequenced and defined the major transcript initiation site. For or Rev: single primer controls; CTRL refers to the no cDNA control. **B**. Sequence of the cloned BCL2L11-P1 region; the sequence shaded in grey indicates coverage by 16 independent human ESTs in Genbank. The arrow indicates the transcript initiation site identified in HEK293 cells by 5' RACE. The red box indicates the sequence of the reverse primer employed in the 5'RACE experiments.

**Figure 4:**
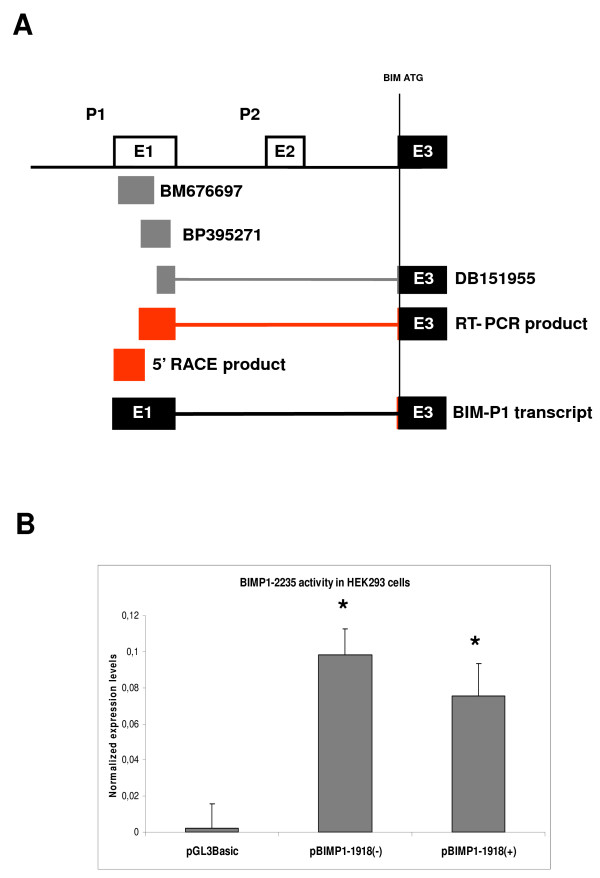
**A**. Schematic summary of results. The human BCL2L11(BIM) locus is shown aligned with relevant ESTs and with the RT-PCR and 5'RACE products. For simplicity the alias BIM is used in the figure instead of the approved gene symbol BCL2L11. The transcript originating in the BIM promoter 1 region defines a novel BIM exon comprising sequences covered by Genbank ESTs and spliced onto the first coding exon of BIM. **B**. BIM-P1 sequences can drive transcription of a luciferase reporter when transiently transfected in HEK293 cells. pBIM-P1 constructs (-1918 relatively to the HEK293 transcript initiation site) demonstrate that the sequence can drive transcription of the reporter in an orientation-independent manner. *p < 0.01, Anova: single factor.

In order to further investigate genomic sequences of the human BCL2L11 locus with promoter activity, we first applied a bioinformatics approach to identify clusters of human ESTs with comparable 5' ends upstream of the first coding exon of the human BCL2L11 locus, informative of the possible existence of distinct transcript initiation sites. We analyzed 13kb of human BCL2L11 genomic sequence immediately upstream of the first BCL2L11 coding exon by BLAST against the Genbank human EST database, and identified 3 clusters with identical/comparable 5'ends (Fig. 1A). The largest group (Group 2; 16 ESTs) corresponded well to the human BCL2L11 promoter already characterized (ca. 3 kb of genomic sequence upstream of the BCL2L11 translational initiation codon), and conserved between human and rat BCL2L11 loci [2,3]. A second, more heterogeneous group (Fig. 1A; Group 3; 12 ESTs) localized to a region comprising sequences described in the rat as potentially harbouring 2 alternative promoters (ca. 1–2 kb upstream of the BCL2L11 translational initiation codon) [10]. A third group (Fig. 1A; Group 1; 22 ESTs) localized to a region ca. 5.5 kb upstream of the ATG, which did not correspond to known BCL2L11 locus sequences in either man or rodents. This group could be subdivided into two subgroups (Fig. 1B; Group 1, subgroup A: 6 ESTs and Group 1, subgroup B: 16 ESTs), each with identical or nearly identical 5' ends, apparently running in opposite directions and potentially suggestive of the existence of a bidirectional promoter. The first indication of the likely existence of an as yet uncharacterized BCL2L11 promoter associated with novel BCL2L11 untranslated exon sequences came from the presence, within ESTs Group 2, of one EST (DB151955) which clearly comprised the first BCL2L11 coding exon (here called Exon 3), but not the first known BCL2L11 untranslated exon (here called Exon 2; Fig. 1B). Instead, novel untranslated exon sequences 5' of the first known untranslated BCL2L11 exon were present, which overlapped with Group 1 subgroup B ESTs. This was suggestive of the presence of a novel BCL2L11 promoter and associated untranslated exon (here called P1 and Exon 1) 5' of the most upstream known BCL2L11 promoter (here called P2 and Exon 2). We therefore focused on Group 1 subgroup B ESTs, as these might represent novel BCL2L11 transcribed sequences by virtue of their overlapping with EST DB151955, which comprises the first BCL2L11 coding exon (Exon 3). Their experimental association with the first BCL2L11 coding exon (Exon 3) in a transcribed sequence would in fact confirm the existence of a new BCL2L11 promoter and associated untranslated exon. Therefore, a series of RT-PCR experiments were performed where the forward primer was anchored within Group 1 subgroup B ESTs and the reverse primer was anchored within the first coding exon of the BCL2L11 locus, downstream of the translational initiation codon (primers indicated by arrowheads in Fig. 1B). Complementary DNA (cDNA) derived from HEK293 mRNA was used in these experiments, as these cells were shown to express BCL2L11 transcripts (this manuscript and data not shown). The sequence of the resulting amplicons (Fig. 2A) confirmed the presence of BCL2L11 coding sequences in the amplified cDNA (Fig. 2B). The alignment (Fig. 2B) of the obtained RT-PCR product sequences with DB151955 and with representative Group 1, subgroup B ESTs (BM676697 and BP395271) confirmed the co-linearity of these transcribed sequences. This finding confirmed that Group 1, subgroup B ESTs indeed comprise novel untranslated BCL2L11 sequences, thus identifying a novel BCL2L11 untranslated exon (now called Exon 1) and confirming the existence of at least one novel putative BCL2L11 promoter (P1) upstream of the previously characterized human BCL2L11 promoter sequences ([3]; here defined P2). Having ascertained that Group 1, Subgroup B ESTs correspond to bona fide BCL2L11 transcripts, we next sought to determine the 5' end of such transcripts in order to determine a putative transcript initiation site, defining a possible 5' exon boundary and a starting point for the definition of a promoter region for P1. 5'RACE experiments performed on HEK293 cells, using a reverse primer anchored within the novel human BCL2L11 exon (Exon 1) and overlapping with the forward primer used in the RT-PCR studies described above, identified a sequence around the 5' end of Group 1, subgroup B ESTs as the transcript initiation site in HEK293 cells (Fig. 3A and 3B), thus defining the likely 5' boundary of this novel human BCL2L11 exon and confirming the existence of a novel human BCL2L11 promoter. A diagram summarizing the position of ESTs and of the RT-PCR and 5'RACE products relatively to human BCL2L11 genomic sequences is provided in Fig. 4A. As transcripts originating from this region do not contain sequences associated with the exon immediately downstream of the published human BCL2L11 promoter ([3]; here called Exon 2), but splice directly onto the first BCL2L11 coding exon ([3]; here called Exon 3), it follows that the human BCL2L11 locus might comprise at least two alternative promoters (P1, characterized in the present manuscript, and P2) and that alternative splicing occurs in the 5' untranslated exons. A third promoter region (P3) may also exist based on data from the rodent BCL2L11 locus [10]. Next, the capacity of P1 sequences to drive gene expression was tested in a series of reporter assays in HEK293 cells. A human genomic region of 2235 bp region comprising a portion of Exon 1 (including the transcript initiation site, and upstream sequences to position -1918 with respect to the HEK293 Exon 1 transcript initiation site; henceforth defined as BCL2L11-P1-1918) was therefore amplified, cloned, sequenced and subcloned into the promoter-less luciferase reporter vector pGL3Basic in both orientations, to give rise to the reporter constructs pBCL2L11-P1-1918(+) and (-). The constructs were then transiently transfected into HEK293 cells and the resulting luciferase activity compared to basal pGL3Basic activity (background control). The results (Fig. 4B) indicate that P1 sequences can drive gene expression, as measured by luciferase activity, though independently of their orientation. Although orientation-dependent activity is usually considered a hallmark of promoter sequences, examples of bona-fide bidirectional promoters are known (e.g. see [8,17,18]). Therefore, P1 may represent a promoter with potential for bi-directional activity, at least in HEK293 cells. This possibility is also supported by the existence of Group 1, subgroup A ESTs as discussed above (see also Fig. 1B), representing ESTs with similar 5' ends but with apparently displaying sequences which are complementary to the sense (with respect to the BCL2L11 locus) P1 direction and starting within ca. 50 bp of the transcript initiation site of E1. Although the existence of a bi-directional promoter in this genomic region is supported by our experimental data and from the existence of coherent ESTs, further studies (e.g. 5'RACE to experimentally identify the antisense transcript initiation sites of the promoter and 3'RACE to clone the associated transcripts) are required to confirm this possibility. As our interest was on the identification of putative novel BCL2L11 promoter sequences, this line was not pursued further.

### Exon 1 containing transcripts are widely expressed in human tissues

**Figure 5:**
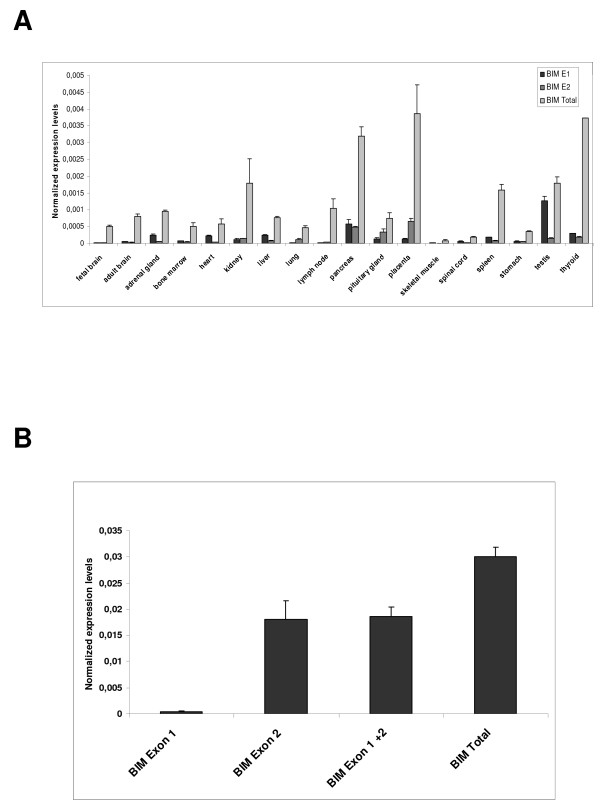
**A**. Real-time expression profiling of BCL2L11(BIM) transcripts as detected with primers specific for Exon 1 (E1), Exon 2 (E2) or capable of recognizing the majority of BCL2L11 transcripts (BCL2L11 total). For simplicity the alias BIM is used in the figure instead of the approved gene symbol BCL2L11. **B**. Expression of BIM P1- and P2-derived transcripts in HEK293 cells, and of BIM transcripts detected by primers capable of recognizing the majority of BIM transcripts (BIM total).

In order to evaluate the possible significance of BCL2L11 transcripts originating from this novel candidate promoter for overall BCL2L11 transcript expression in man, the expression of BCL2L11 mRNAs bearing the alternatively spliced Exon 1 or Exon 2 sequences, or of total BCL2L11 transcripts, was evaluated in a panel of cDNAs derived from human tissues by real-time PCR, using SYBR green technology. Primer pairs specific for BCL2L11 Exon 1 or 2, or for a BCL2L11 transcribed region presumably present in most characterized BCL2L11 transcripts (specific for a 3' region of BCL2L11 coding sequences) were employed. The results indicated that transcripts originating from the candidate novel BCL2L11 promoter P1 were readily detectable in a number of human tissues (Fig. 5A), and contributed most significantly to BCL2L11 mRNA levels in testis, heart and liver (by comparison with BCL2L11 transcript levels detected with primers specific for a 3' region of BCL2L11 coding sequences). Next, the relative contribution of Exon 1- and Exon 2-containing transcripts to total BCL2L11 expression in HEK293 cells was then evaluated by real-time PCR, using SYBR green technology. The results (Fig. 5B) again indicated that BCL2L11 transcripts containing Exon 1 or Exon 2 are expressed in HEK293 cells.

### Exon 1 containing transcripts are coordinately and coherently regulated with other BCL2L11 transcripts by TSA in HEK293 cells

**Figure 6:**
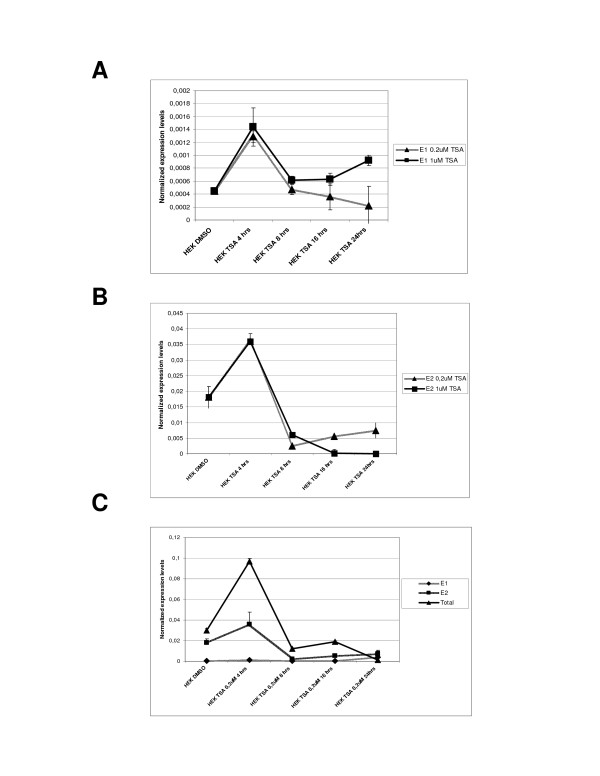
**Endogenous BCL2L11(BIM) transcripts comprising E1, E2 and 3'coding sequences are coordinately regulated by TSA in HEK293 cells**. For simplicity the alias BIM is used in the figure instead of the approved gene symbol BCL2L11. **A**. Time course of expression of Exon 1-containing BIM transcripts in HEK293 cells following TSA treatment as measured by real-time PCR, using an E1-specific primer set (E1); TSA rapidly induces BIM-E1 expression at 4 hrs at both concentrations tested. **B**. The same analysis was performed using primer sets specific for the (previously known) BIM Exon 2; The expression of the P2-derived BIM transcript in HEK293 cells (E2) is induced by TSA at 4 hrs at the concentrations of 0.2 μM (i) and 1 μM (ii), as for E1 containing BIM transcripts. **C**. Time course of expression of BIM transcript levels in HEK293 cells treated with 0.2 μM TSA, and detected using primers specific for a 3' region of the coding sequence (and presumably recognizing most BIM transcripts; Total) or primers specific for Exon 1- or Exon 2-bearing BIM transcripts (E1 or E2, respectively).

The biological significance of the newly identified putative BCL2L11 promoter would be further strengthened if transcripts derived from it could be shown to be co-regulated together with other BCL2L11 transcripts, in a cellular context where BCL2L11 mRNA levels are known to be modulated. In order to investigate this possibility, the levels of different BCL2L11 mRNAs were quantitatively measured in cDNA from HEK293 cells or HEK293 cells treated with a known inducer of BCL2L11 expression in human cells, namely the histone deacetylase inhibitor Trichostatin A (TSA) [15], using the primer pairs mentioned above. In HEK293 cells, Exon 1 containing BCL2L11 transcripts are rapidly and transiently induced by Trichostatin A (TSA; Fig. 6A). In HEK293 cells, BCL2L11 transcripts comprising Exon 2 sequences (and thus derived from P2) are by far the most abundant (approximately 50 times more abundant than Exon 1-containing transcripts; Fig. 6). Interestingly, the steady-state levels of human BCL2L11 transcripts comprising Exons 1 and 2 are rapidly and transiently increased by TSA, confirming the reported regulation of BCL2L11 expression by this inhibitor of histone deacetylases [15]. We next investigated the relative contribution of BCL2L11 transcripts comprising Exons 1 and 2 to total BCL2L11 transcript levels (Fig. 6C). Exon 1-containing transcripts display the same induction profile of transcripts detected by a primer pair presumably recognizing most known BCL2L11 transcripts (Fig. 6A and 6C). Exon 2-containing transcripts account for the majority of steady-state BCL2L11 transcripts in HEK293 cells. In untreated HEK293 cells, the cumulative levels of steady-state BCL2L11 transcripts encoding Exons 1–2 are lower than levels of steady-state BCL2L11 transcripts detected using a primer set potentially recognizing most BCL2L11 transcripts (Fig. 5C), and account for approximately 60% of these. These differences may result from differential stability of BCL2L11 transcripts bearing different 5'ends, or from other as yet uncharacterized reasons such as the presence of further promoters in this locus. Collectively, these expression data support the hypothesis that P1-derived transcripts are a bona-fide human BCL2L11 mRNAs displaying a pattern of regulation which is coherent with the overall regulation of BCL2L11 expression by a known small molecule modulator, at least in HEK293 cells.

### E2F regulates the activity of BCL2L11-P1

**Figure 7:**
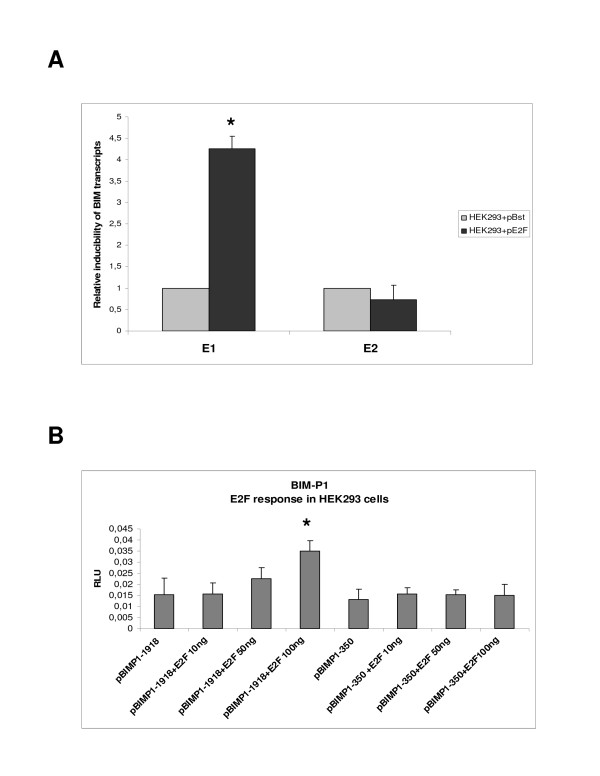
**BCL2L11(BIM)-P1 activity is regulated by E2F**. For simplicity the alias BIM is used in the figure instead of the approved gene symbol BIM. **A**. Inducibility of endogenous E1 and E2-bearing BIM transcripts by transiently transfected E2F expression construct in HEK293 cells; real-time experiments were performed as previously in cells transfected with either pBst or an E2F expression construct, and the fold inducibility (relatively to pBst transfected cells) of individual transcripts by the E2F expression plasmid is presented; the results confirm reporter data and show that the expression of endogenous BIM transcripts derived from P1 can be induced by E2F. *p < 0.01, Anova: single factor. **B**. Analysis of the effects of E2F overerexpression on pBIM-P1-1918(+) activity in HEK293 cells. A concentration-dependent inducibility of P1 activity is observed; by contrast, a version of P1 comprising a 5' deletion of 1300 bp results in a promoter construct with a basal activity comparable to P1-1918, but without the capacity for E2F-mediated activation, suggesting the presence of E2F responsive elements in the deleted region. *p < 0.01, Anova: single factor.

**Figure 8:**
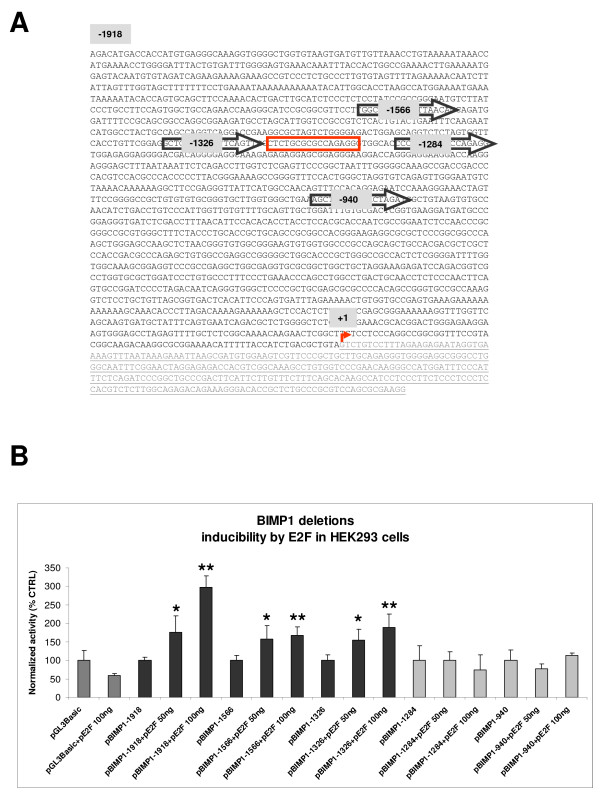
**Effects of E2F on BCL2L11-P1 constructs**. For simplicity the alias BIM is used in the figure instead of the approved gene symbol BCL2L11. **A**. Sequence of the -1918 P1 region showing the position of 5' deletion variants. Forward primers used to generate the various P1 5' deletion variants are indicated (arrows). The putative E2F binding site is boxed, and the transcript initiation site is indicated (+1). **B**. Analysis of the effects of E2F over-expression on pBIM-P1 and 5'deletion variants activity in HEK293 cells. A concentration-dependent inducibility of P1 activity is observed in construct pBIM-P1-1918, -1565 and -1326, which contain a putative E2F responsive element. By contrast, pBIM-P1-1284 and -940 display basal activities comparable to pBCL2L11-P1-1918, but without the capacity for E2F-mediated response, suggesting a loss of E2F responsive element(s). *p < 0.05 and **p < 0.01, Anova: single factor.

**Figure 9:**
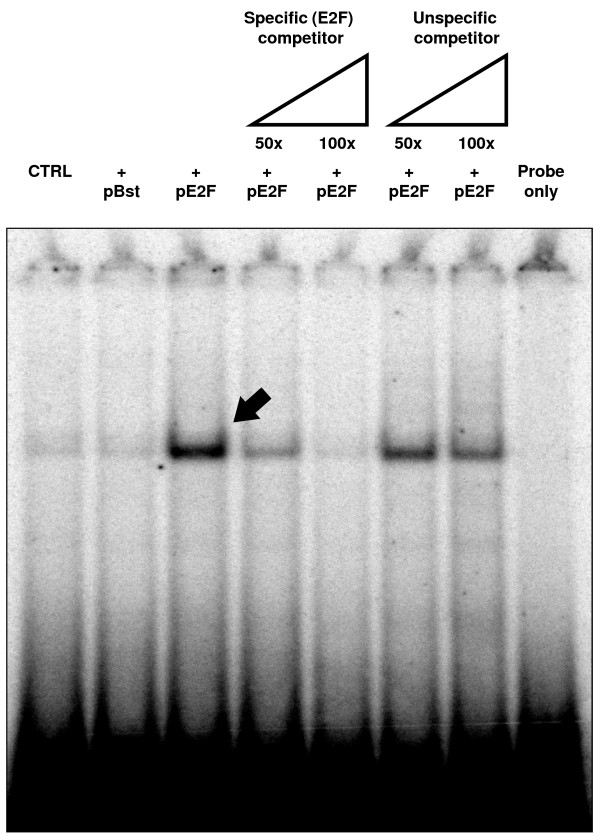
**E2F binding to a candidate E2F binding site in BCL2L11-P1**. EMSA on the candidate E2F binding site (CTCTGCGCGCCAGAGG). HEK293 cells were transfected with the E2F expression construct, and nuclear extracts were assayed for capacity for specific binding to the candidate E2F binding site through competition with a specific (E2F) or unspecific (NF-κB) unlabelled competitor double stranded (ds) oligonucleotide. A specific shift is identified (arrow) upon transfection with the E2F expression plasmid, which can only be competed with a specific ds oligonucleotide.

E2F is a known regulator of BCL2L11 transcription during apoptosis [2,11], as evidenced by studies performed on the rat and mouse BCL2L11 loci. In order to determine if E2F can regulate the expression of human BCL2L11 promoters in HEK293 cells, the real time primers employed above were used to measure levels of BCL2L11 transcripts originating from the two known human promoters (namely the novel promoter BCL2L11-P1 described here and from the previously known promoter BCL2L11P2) in response to transient transfection of a commercially available human E2F expression construct. This was made possible by the high transfection efficiency achieved in HEK293 cells with standard lipofection-mediated methodologies (>80%; data not shown). As indicated in Fig. 7A, overexpression of E2F results in a significant increase in the steady-state levels of Exon 1-comprising transcripts, suggestive of a role for E2F in regulating the transcription, processing or stability of endogenous P1-derived transcripts. In contrast, Exon 2-containing transcripts were not affected, suggesting that the endogenous P2 is not significantly modulated by E2F under these experimental conditions. As the P2 promoter is regulated directly or indirectly by E2F in rodents [2,11], our results suggest that this regulation may not be conserved in humans, at least under these experimental conditions and in HEK293 cells. In order to determine if E2F could directly regulate P1 activity, we next investigated the capacity of the E2F expression plasmid to modulate the activity of BCL2L11-P1-1918 (Fig.7B). We observed an induction of reporter activity with BCL2L11-P1-1918 which was dependent on the concentration of E2F expression plasmid. Conversely, we did not observe such E2F inducibility with a 5' deletion variant of P1 lacking all but 350 bp of the genomic region upstream of the transcript initiation site (BCL2L11-P1-350). These data suggested that an E2F-responsive regulatory element is located within P1, between position -1918 and -350 bp. We therefore searched for candidate E2F binding sites in the BCL2L11-P1-1918 sequence using a web-based *in silico *algorithm, and identified a potential E2F element at position -1309 (Fig. 8A). A number of 5' deletion variants of BCL2L11-P1-1918 were therefore generated by proofreading PCR using the indicated forward primers (Fig. 8A), and subsequently cloned into pGL3Basic to generate the reporter constructs BCL2L11-P1-1566, -1326, -1284, -940. These constructs were then tested for regulation by overexpressed E2F in HEK293 cells, as described above. The results (Fig. 8B) indicated that significant E2F inducibility was lost upon deletion of a region of only 42 bp, located between positions -1326 and -1284, and comprising the candidate E2F binding site located at position -1309. Direct proof of the interaction between E2F and this candidate E2F binding site was therefore sought by electromobility shift assays (EMSA). A double stranded oligonucleotide corresponding to the candidate E2F binding site was labeled and its capacity to bind E2F was studied using nuclear extracts from HEK293 cells transiently transfected with the human E2F expression construct. Competitions with cold double stranded oligonucleotides comprising either a canonical E2F binding site or a binding site for an irrelevant transcription factor (NF-κB) were used in order to determine the specificity of the observed interaction. As shown in Fig. 9, a strong bandshift (arrow) is observed using nuclear extracts from HEK293 cells transfected with the E2F expression plasmid. A much weaker but identically migrating band is also visible in nuclear extracts from untransfected HEK293 cells or from cells transfected with a control plasmid. Competition with excess specific or unspecific competitors indicates that the observed band is specifically competed by an oligonucleotide comprising a canonical E2F binding site (Fig. 9). Therefore, the genomic element identified in BCL2L11-P1 at position -1309 as a potential E2F binding site is indeed a functional E2F binding site and provides a molecular basis for E2F regulation of BCL2L11-P1.

## Discussion

We have identified a novel putative promoter and associated exon of the human BCL2L11 locus in HEK293 cells and showed regulation of this promoter by the transcription factor E2F, which is known to modulate BCL2L11 transcription in other species. BCL2L11 transcripts derived from this promoter are widely expressed in human tissues, and contribute significantly to BCL2L11 expression in testis, heart and liver. The real-time PCR data presented here constitutes the first systematic, quantitative analysis of steady-state BCL2L11 transcript expression levels in different human tissues. Overall, the data obtained on the distribution of BCL2L11 mRNA levels in different human tissues correlate with published data on the distribution of BCL2L11 RNA and protein in human tissues, which indicates relatively higher expression in testis and spleen [23,24], organs where BCL2L11 has recognized functions in spermatogenesis and hematopoiesis, respectively [25,26]. Interestingly, the analysis presented in this manuscript indicates that the highest expression levels of BCL2L11 transcripts were detected in pancreas, placenta and thyroid tissue, suggesting an important role for BCL2L11 in the normal physiology of these tissues. With respect to P1-derived transcripts, up to 70% of total BCL2L11 transcripts are contributed from the newly identified promoter in the testis, an organ where BCL2L11 activity was shown to contribute critically to spermatogenesis [25], suggestive for a prominent role for the putative BCL2L11 P1 in the expression of BCL2L11 in this tissue. Although the biological significance of BCL2L11 P1 remains to be determined, the expression of P1-derived transcripts in various human tissues of relevance to BCL2L11 biological function argues for a physiological significance of such transcripts. The biological significance of the newly identified putative BCL2L11 promoter was strengthened by the observation that transcripts derived from it could be shown to be co-regulated together with other BCL2L11 transcripts by a known pharmacological modulator of BCL2L11 expression (TSA), in the same cell line where such transcripts have been identified. Another piece of circumstantial evidence pointing to a physiological role for BCL2L11 P1 in BCL2L11 expression is its regulation by the transcription factor E2F. Studies performed on the rodent BIM promoters (the equivalents of BCL2L11 P2) have identified this transcriptional regulator as a modulator of BCL2L11 expression during apoptotic processes [2,11]. Through a combination of reporter assays and DNA-protein interaction studies, we were able to demonstrate that E2F can upregulate BCL2L11 P1 activity in HEK293 cells through a cis-acting element identified through a bioinformatics analysis, which can bind E2F in EMSA assays and whose presence is required for responsiveness to overexpressed E2F. This is the first indication of direct transcriptional regulation of a BCL2L11 promoter by E2F, as previous reports of E2F involvement in the regulation of BCL2L11 expression have shown either an indirect regulation through other transcription factors [2] or have not investigated the underlying mechanism [11]. The potential contribution of P1 activity during BCL2L11-mediated apoptotic processes induced by E2F may therefore be physiologically important and deserves further investigation. A number of further aspects are worthy of further analysis. First, further characterization of BCL2L11 P1 in different cellular contexts/backgrounds is required, in particular in contexts where BCL2L11 expression is higher than that observed in HEK293 cells where BCL2L11 expression is relatively low, in order to confirm that such new putative promoter is indeed generally active and drives BCL2L11 transcripts in most situations where the locus is transcribed. Second, the relative contribution of P1- and P2-derived transcripts to BCL2L11 protein expression may differ and needs to be investigated, as E1 and E2 are alternatively spliced and may contribute differently to mRNA stability and translation. Finally, it remains to be determined if a homologue of BCL2L11-P1 exists in rodents. Interestingly, a BLAST analysis using 13 kb of mouse genomic sequences upstream of the first coding exon of the murine BCL2L11 locus revealed a similar pattern of ESTs as that evidenced in this manuscript for the human locus, suggesting the potential existence of a BCL2L11-P1 homologue in mice (data not shown) and providing further indirect evidence of an important physiological role for P1 in BCL2L11 expression.

## Conclusion

Through a combined bioinformatics and experimental approach we have identified a novel putative promoter and associated untranslated exon in the human BCL2L11 genomic locus, upstream of the previously characterized promoter. This candidate promoter generates transcripts which are widely expressed in human tissues, and which contribute significantly to overall BCL2L11 expression in tissues where BCL2L11 is known to play important physiological roles, such as the testis. Our data are suggestive of a complex pattern of transcriptional regulation of the human BCL2L11 locus, comprising alternative promoter usage and alternatively spliced 5' untranslated exons, and suggests the presence of additional, as yet unidentified promoter(s). Although, the general relevance and impact of BCL2L11-P1 and of its regulation by E2F in other human cell lines or in vivo remains to be determined, the present study offers novel insights into the structure and regulation of the human BCL2L11 locus.

## Methods

### Bioinformatics

Bioinformatics analyses were performed using tools available at NCBI [28]. E2F binding site prediction was performed at GBF [29].

### DNA and RNA extraction, cDNA synthesis and PCR amplification

Genomic DNA was isolated from HEK293 cells as described [19]. Total RNA was isolated using a commercial kit (RNeasy Kit, Quiagen, Hilden, Germany) as per manufacturer's instructions. RNAs from human tissues were obtained from a commercial source (Clontech Inc., CA). cDNA was synthesized from 2 μg total RNA using Superscript II Reverse Transcriptase (Invitrogen) as per manufacturer's instructions, using oligodT as a primer. Preparative PCR was performed using Phusion DNA polymerase from Finnzymes Inc (Espoo, Finland), as per manufacturer's instructions.

### Cloning and generation of plasmid constructs

All cloning and subcloning was performed using PCR approaches as described in the text. For cloning and transformation, One Shot TOP10 Chemically Competent E. coli, Zero Blunt PCR Cloning Kit were from Invitrogen (San Diego, CA) and were used as per manufacturer's instructions. DNA manipulations were performed using techniques essentially as described [19]. All PCR reactions were performed using a proofreading polymerase (Phusion polymerase, Finnzymes Inc.). Human genomic sequences associated with the BCL2L11 locus were amplified from HEK293 genomic DNA using a specific primer set and a nested PCR strategy (BCL2L11-P1-1918-FOR1 TAACAACATCAGTGCGGCTC and BCL2L11-P1-1918-REV1 AGAGAACGCAGTGTGAGAAG, followed by a second round using BCL2L11-P1-1918-FOR2 AAGAGCTCGGCTCAGACATCA and BCL2L11-P1-1918-REV2 AAAAGCTTCACCTCCTTCGCG). A 2235 bp fragment comprising the genomic region of interest was subcloned into pGL3Basic (Promega Inc.) as a transcriptional fusion (BCL2L11-P1-1918). Deletions construct of BCL2L11-P1-1918 were prepared using following primer set BCL2L11-P1-1566 FOR AAGAGCTCGGCATTGGCGTTAACAGC, BCL2L11-P1-1326 FOR AAGAGCTCGCTCTTCCTGTTCAGTTC, BCL2L11-P1-1284 FOR AAGAGCTCCCCAGGCACTCCAGAGGT, BCL2L11-P1-940 FOR AAGAGCTCAGCTGCTGTCACTAGATG and for all reactions BCL2L11-P1-2000 REV AAAAGCTTCTCCCCACCCTCT. RT-PCR to confirm the transcript originating from BCL2L11-P1 region was performed using the following primer set HsBCL2L11E1-FOR1 TTCAGCACAAGCCATCCTCC and HSBCL2L11-CDS-REV1 ACCTCCGTGATTGCCTTCAG. All constructs were confirmed by DNA sequencing.

### RT-PCR and RACE cDNA cloning

RT-PCR and RACE-PCR was performed essentially as described previously [4]. First strand synthesis was performed using Superscript II according to manufacturer's instructions, employing 2 μg total RNA. 5' Rapid amplification of cDNA ends (RACE) was carried out by reverse transcription of HEK 293 cells polyadenilated (poly A) RNA employing the primer, CCAGTGAGCAGAGTGACGAGGACTCGAGCTCAAGCTTTTTTTTTTTTTTTT, which contains an anchor region. For RT-PCR, a forward primer anchored to sequences present within Group 1, subgroup A ESTs (BCL2L11-RT-FOR AGCACAAGCCATCCTCCTTC) and a reverse primer anchored within the first BCL2L11 coding exon, 3' to the translational initiation codon (BCL2L11-RT-REV TTGCCTTCAGGATTACCTTG) were employed. For RACE-PCR, a nested amplification approach was used, employing the following primer BCL2L11-P1-RACE1 AGAGAACGCAGTGTGAGAAG and the 5' anchored externally primer RACEA CCAGTGAGCAGAGTGACG. Conditions were as follows: annealing 62°C for the first 10 cycles, then 40 cycles at 52°C (Dyad DNA Engine, Biorad, USA). For second round the following primer set was employed: BCL2L11-P1-RACE2 GGAGGATGGCTTGTGCTGAA and the primer anchored internally to RACEA, of sequence GAGGACTCGAGCTCAAGC using the same amplification program of the first round. The amplicon was loaded on a 1.5% gel, purified, and finally cloned. Purification was done using PCR purification kit (Quiagen, Hilden, Germany) and cloning in Zero Blunt cloning kit. Sequences were confirmed by sequencing both strands of more than one clone (MWG, Martinsried). All primer sets yielding PCR products from cDNA templates were tested for the absence of products resulting from amplification using the corresponding RNA as a template, to detect eventual problems due to genomic DNA contamination (data not shown).

### Cell culture

Human Hembrional Kidney cells (HEK293) were provided from ATCC and were grown at 37°C, 5% CO_2 _in DMEM, medium (Gibco) supplemented with 10% fetal bovin serum (Biowhittaker, Cambrex), 1% streptomycin ampicillin (Cambrex) and 1% glutamax (Gibco). Trichostatin A was from Sigma Chemical Company. A 10 mM solution of TSA in dimethylsulphoxide was prepared and stored a -20°C until use. HEK293 plated in 6 wells were treated the day after with TSA 0.2 μM or 1 μM for 4, 8, 16, 24 hours. Control cells were treated with dimethylsulphoxide. At the end of incubation period cells were harvested. Total RNA extraction and cDNA synthesis were performed as described before.

### Transient transfections and luciferase assays

The human E2F expression construct was purchased from a commercial source (Origene). DNA was prepared with Plasmid Maxi kits (Qiagen, Hilden, Germany). HEK293 cells were cotransfected with 0.05 μg of reporter plasmid, 0.01 μg of Renilla vector (pRL-TK, Promega), 0.01 μg of empty plasmid Bluescript (Stratagene, Inc.) or 0.1 or 0.05 μg Human E2F expression construct (Origene, Inc.) in 95 μl of serum-free medium per well in 96-well plate using Lipofectamine 2000 [5]. Three hours later, medium with lipofectamine 2000 was replaced with fresh complete medium. Before use, 96 well plates were polylisinated with poly-D-lysine. Luciferase assays were performed using the Dual Reporter System (Promega Inc.). 24 hours post-transfection, cells were lysed in the buffer provided in the Promega Luciferase System. Dual luciferase assays were performed according to the manufacturer's instructions, using a Mithras LB 940 luminometer (Berthold). Relative luciferase activities were obtained by normalizing the luciferase activity against Renilla luciferase activity.

### Real Time PCR

Quantitative real-time PCR (qPCR) primers specific for individual BCL2L11 exons and for total BCL2L11 transcripts used were BCL2L11E1-FOR CCATGATTTCCCATTTCTCA, BCL2L11E1-REV GGCTTGTGCTGAAAGAAACA, BCL2L11E2-FOR GCCACTACCACCACTTGATTCTT, BCL2L11E2-REV AACCGAATACCGCGATGATG, BCL2L11-1947F (5'-TGGATATTGTCAGGCCACTTG-3') and BCL2L11-2018R (5'-CATAAGGAGCAGGCACAGAGAA-3'). For normalization of Quantitative real-time PCR, β-actin levels were determined in each sample using the following primers: β-actin-FOR CCTGGCACCCAGCACAAT and β-actin-REV GCCGATCCACACGGAGTACT. Real time quantitative PCR analysis was carried out using an iQ5 cycler machine (Biorad). Reactions were prepared in triplicate using 2× SYBR Green Supermix (Biorad) according to manufacturer's instructions to a final volume sample of 30 μl. The program used was the following: 95°C 3' and 45 cycles at 95°C/30" and 55°C/30". For each experiment, at least 2 independent electrophoresis runs were performed.

### Electrophoretic mobility shift assay (EMSA)

HEK293 were plated in 6 well plates and the day after were transfected as describe before using 2.5 μg of E2F plasmid or 2.5 μg of Bluescript plasmid. 24 hrs post-transfection, cells were washed twice with PBS and harvested for nuclear protein extraction according to the method of Osborn et al. [16] with minor modifications. The protease inhibitors leupeptin (10 μg/ml), antipain (5 μg/ml), and pepstatin (5 μg/ml) and phenylmethylsulfonyl fluoride (1 mM) were added. Protein concentration was measured by the Coomassie assay (Pierce), using BSA as standard protein. Binding reactions were carried out in a final volume of 20 μl using 8–10 μg nuclear extract according to the conditions already specifically described for E2F1 binding site [20]. Radiolabelled probe was added last to each reaction mixture and samples were incubated at room temperature for 30 min. [7]. In a competition assays a 50–100 × fold molar excess of unlabeled double-stranded oligonucleotide was added to the reaction mixture. Samples were then loaded onto a 5% (30:1:2) native polyacrylamide gel in 45 mM Tris Base, 45 mM Sodium borate, 10 mM EDTA ph 8 (0,5 × TBE) and run at 150 V, dried, and exposed to Storage Phosphor Screen (Amersham Biosciences). After overnight exposition, the image was visualized by PhosphorImager SF (Molecular Dynamics, Inc). Double stranded oligonucleotides were synthesized, annealed to complementary synthetic oligonucleotide in vitro and radiolabeled using T4 polynucleotide kinase (Boeheringer) and [γ-32P] labelled ATP (Perkin-Elmer, Inc.). Unincorporated nucleotides were removed by Sephadex G-50 column chromatography [19]. The following pairs of oligonucleotides were employed (E2F sites underlined) E2F-1 P1 5'-AGTTCCCTCTGCGCGCCAGAGGGTG-3'. As cold competitor were used the following oligonucleotids: E2F-1 C+ 5'-ATT TAAGTTTCGCGCCCTTTCTCAA-3', E2F-1 P1 5'-AGTTCCCTCTGCGCGCCAGAGGGTG-3' and NF-κB 5'-AATGTGGGATTTTCCCATG-3'.

## Authors' contributions

All authors have read and approved the final manuscript. MG performed most of the experimental work towards all figures; AC contributed Fig. 1 and supervised the experimental work except for Fig. 9; DD contributed experiments towards Fig. 5, MDa, CC, MDo contributed experiments towards Fig. 9 and AS and MP provided the original concept for the experimental work described and wrote the manuscript.
